# Heterologous Expression and Characterization of a High-Efficiency Chitosanase From *Bacillus mojavensis* SY1 Suitable for Production of Chitosan Oligosaccharides

**DOI:** 10.3389/fmicb.2021.781138

**Published:** 2021-11-29

**Authors:** Jianrong Wang, Xiaoming Li, Hao Chen, Bilian Lin, Liangzhong Zhao

**Affiliations:** ^1^College of Food and Chemical Engineering, Shaoyang University, Shaoyang, China; ^2^Hunan Provincial Key Laboratory of Soybean Products Processing and Safety Control, Shaoyang, China; ^3^Bioengineering Research Center, Guangzhou Institute of Advanced Technology, Guangzhou, China

**Keywords:** *Bacillus mojavensis*, chitosanase, high efficiency, *Pichia pastoris*, Chitosan oligosaccharides

## Abstract

Chitosanase plays an important role in enzymatic production of chitosan oligosaccharides (COSs). The present study describes the gene cloning and high-level expression of a high-efficiency chitosanase from *Bacillus mojavensis* SY1 (CsnBm). The gene encoding CsnBm was obtained by homologous cloning, ligated to pPICZαA, and transformed into *Pichia pastoris* X33. A recombinant strain designated X33-C3 with the highest activity was isolated from 120 recombinant colonies. The maximum activity and total protein concentration of recombinant strain X33-C3 were 6,052 U/ml and 3.75 g/l, respectively, which were obtained in fed-batch cultivation in a 50-l bioreactor. The optimal temperature and pH of purified CsnBm were 55°C and 5.5, respectively. Meanwhile, CsnBm was stable from pH 4.0 to 9.0 and 40 to 55°C. The purified CsnBm exhibited high activity toward colloidal chitosan with degrees of deacetylation from 85 to 95%. Furthermore, CsnBm exhibited high efficiency to hydrolyze different concentration of colloidal chitosan to produce COSs. The result of this study not only identifies a high-efficiency chitosanase for preparation of COSs, but also casts some insight into the high-level production of chitosanase in heterologous systems.

## Introduction

As the second abundant polysaccharide on earth, chitin is composed of N-acetylglucosamine linked by β-1,4 glycoside bonds, and the lack of solubility limits its industrial application (Kaczmarek et al., 2019; Schmitz et al., 2019). Chitosan, the derivative products from deacetylation of chitin, exhibits better commercial value than chitin owing to its better solubility (Romanazzi et al., 2018). Chitosan is soluble in diluted acid and applied in some fields, but high viscosity and poor water solubility limit further industrial application. Chitosan oligosaccharides (COSs) are oligomers with the degree of polymerization (DP) 2-20 and mainly from hydrolysis of chitosan. Compared with chitin or chitosan, COSs have many advantages such as excellent water solubility, low viscosity, and biodegradability (Li et al., 2016; Yuan et al., 2019). Meanwhile, many researches demonstrated that COSs display various bioactivities such as anti-tumor (Xu et al., 2017), positive effects on host gut health (Zhang et al., 2018; Wu et al., 2019), and immune activation (Kim et al., 2006). Because of the excellent water solubility and various biological activities, COSs have attracted increasing interest in many industries. The preparation methods of COSs mainly include chemical, physical, and enzymatic method. Among these three methods, the enzymatic production of COSs has received more attention due to its environmental friendliness, high identification of hydrolysis sites, non-toxicity, and controllability (Yuan et al., 2019). However, the high production cost limits the enzymatic method as the main choice for industrial-scale preparation of COSs (Yuan et al., 2019). Therefore, the isolation and high-level expression of high-efficiency chitosan degrading enzymes can provide a basis for the enzymatic production of COSs.

Chitosanases belong to glycoside hydrolase (GH) and catalyze hydrolysis of β-1,4 glycoside bonds of chitosan to produce COSs. Based on the sequence similarity, chitosanases are grouped into seven GH families by CAZy database, which include GH3, GH5, GH7, GH8, GH46, GH75, and GH80. Among these seven GH families, chitosanases from GH46 have been characterized extensively (Viens et al., 2015). The GH46 chitosanases are mainly derived from bacterial sources, especially from *Bacillus* and *Streptomyces* (Viens et al., 2015). As an important member of GH46, chitosanases from *Bacillus* exhibit excellent catalytic properties and the end products of chitosan hydrolyzed by *Bacillus* chitosanases are mainly composed of chitobiose, chitotriose, and chitotetraose (Qin et al., 2018b; Yang et al., 2020; Cui et al., 2021). Chitosanases from *Bacillus* exhibit great potential value to preparation of COSs, but low-level production of chitosanases in wild-type strain limits further industrial-scale application (Thadathil and Velappan, 2014). Therefore, it is crucial to improve the production of *Bacillus* chitosanases. Heterologous expression is an effective method to improve the production of recombinant proteins. For chitosanases, the most used host is *Escherichia coli* (*E. coli*), followed by *Pichia pastoris* (*P. pastoris*). The results of previous studies demonstrated that *P. pastoris* is more suitable for production of chitosanases than *E. coli* (Peng et al., 2013; Ding et al., 2019; Zhou J. L. et al., 2020; Zheng et al., 2021). Compared with *E. coli*, *P. pastoris* has many advantages such as high expression level, extracellular secretion of target recombinant protein, low secretion level of endogenous protein, and mature fermentation process. Therefore, high-level expression of chitosanases in *P. pastoris* could cut the cost and provide foundation for its application in industrial-scale production of COSs.

In this study, a high-efficiency chitosanase (named CsnBm) was cloned from *Bacillus mojavensis* SY1 (*B. mojavensis* SY1) and expressed in *P. pastoris*. The properties of purified CsnBm were characterized. Furthermore, hydrolytic pattern and preparation of COSs were investigated. The results of this study will provide a high-efficiency chitosanase for production of COSs.

## Materials and Methods

### Materials

The *P. pastoris* X33, vector pPICZαA, and zeocin were purchased from Invitrogen (Carlsbad, CA, United States). The *E. coli* strain Top 10 was used to propagate plasmids. *B. mojavensis* SY1 was isolated from shrimp and crab shell waste and conserved in our laboratory. Restriction enzymes (*Eco*RI, *Sac*I, and *Xba*I), T_4_-DNA ligase, and DNA polymerase (PrimeSTAR^TM^ HS) were purchased from Takara Biotechnology (Beijing, China). Endo-glycosidase H_*f*_ (Endo H_*f*_) was purchased from New England Biolabs (NEB, Ipswich, MA, United States). TIANprep Midi Plasmid and TIANamp Bacteria DNA Kit were purchased from Tiangen Biotech (Beijing, China). Powdery chitosan with 85 to 95% degrees of deacetylation (DA) and glucosamine were purchased from Yuanye Biotechnology (Shanghai, China). Chitobiose, chitotriose, chitotetraose, chitopentaose, and chitohexaose were obtained from Long Dragon Bio (Huizhou, China). Furthermore, GlcN, (GlcN)_2_, (GlcN)_3_, (GlcN)_4_, (GlcN)_5_, and (GlcN)_6_ were short for glucosamine, chitobiose, chitotriose, chitotetraose, chitopentaose, and chitohexaose, respectively.

Media for *E. coli* and *P. pastoris* include LBZ (LB with 25 μg/ml zeocin), YPDZ (yeast extract peptone dextrose medium with 100 μg/ml zeocin), BMGY (buffered glycerol complex medium), and BMMY (buffered methanol complex medium). Media for high cell density fermentation was BSM. LBZ, YPDZ, BMGY, BMMY, and BSM were prepared according to a previous study (Wang et al., 2013).

### Gene Cloning and Bioinformatics Analysis

Genomic DNA from *B. mojavensis* SY1 was extracted by using the TIANamp Bacteria DNA Kit. Two primers (*csnbm*-fw, 5′-ATGAAAATCAGTTTGGAGAAA-3′ and *csnbm*-rev, 5′-TTATTT GATTACGAAATCACC-3′) based on the sequence of *B. mojavensis* strain UCMB5075 chromosome (GenBank: CP051464.1, 1279715-1280551) were designed for PCR amplification. The cloned fragment was ligated into the pMD20T vector and then sequenced. The obtained sequence was first analyzed *via* online BLASTn and BLASTx provided by the National Center for Biotechnology Information (NCBI). Sequence identity of CsnBm against different chitosanases was performed by DNAman 6.0. The signal peptide was analyzed by SignalP 5.0 server. Modeler 10.1 was used for homology modeling of CsnBm and the crystal structure of chitosanase from *Bacillus subtilis* (*B. subtilis*) MY002 was chosen as template (PDB deposition: 7C6D). PyMOL was used to analyze the obtained model.

### Heterologous Expression of CsnBm in *Pichia pastoris* X33

The process for heterologous expression of CsnBm in *P. pastoris* X33 was the same as previous studies (Peng et al., 2013; Wang et al., 2019b). Two primers were designed (*csnbm*-*Eco*RI-fw, 5′-AGCGAATTCGCCGGACTGAACAAGGATCAA-3′ and *csnbm*-*Not*I-fw, 5′-AGCTGGCGGCCG CTTTGATTACGAAA TCACCGT-3′) for PCR amplification. The resulting PCR product without the native signal sequence was cloned into the *P. pastoris* expression vector pPICZαA at *Xba*I and *Eco*RI sites to generate pPICZαA-*csnbm*. The expression vector pPICZαA-*csnbm* was linearized with *Sac*I and electrotransformed into *P. pastoris* X33 competent cell. After transformation, cells were screened on YPDZ plates with different concentrations of zeocin (from 100 to 500 μg/ml). The method for screening transformants was the same as the previously described method (Wang et al., 2019a). The detailed protocol is provided in the Supplementary Material.

The recombinant strain with the highest activity was further cultivated in 7- and 50-l bioreactors. The cultivation conditions and medium composition of high cell density fermentation are provided in the Supplementary Material. The enzyme activity, cell density (wet cell weight), and total protein concentration were monitored throughout the fermentation. The chitosanase activity was measured according to the previous methods (Guo et al., 2019). Chitosan (0.5 g) with 90% DA was dissolved in 100 ml of acetic acid-sodium acetate buffer (pH 5.5, 0.2 mM) and used as substrate. After 2 min preheating at 55°C, 50 μl of diluted enzyme was added to 350 μl 0.5% (w/v) colloidal chitosan. In addition, the reaction mixture was incubated at 55°C for 10 min, and then 600 μl of 3,5-dinitrosalicylic acid (DNS) was added to end the reaction. The reducing sugars released from the substrates were determined with DNS method. One unit of enzyme activity was defined as the amount of enzyme that releases 1 μmol reducing sugars/min. The concentration of total protein was detected by the Bradford method using BSA as standard. Wet cell weight (WCW) was obtained by centrifuging 10-ml samples in a pre-weighted centrifuge tube at 8,000 × *g* for 10 min and discarding supernatant.

### Purification, Substrate Specificity, and Kinetic Parameters

The method for purification of recombinant CsnBm was the same as the previously described method (Wang et al., 2015). The supernatant without cell was harvested by centrifuging fermented broth at 10,000 × *g* for 10 min at 8°C, and then concentrated by ultrafiltration with a membrane of 10 kDa cutoff. The supernatant containing recombinant CsnBm was purified by Ni^2+^-nitrilotriacetate (NTA) resin chromatography (Sangon Biotech, China). The purified recombinant CsnBm was analyzed by SDS-PAGE.

For deglycosylation, the purified CsnBm was deglycosylated using 300 U of Endo H_*f*_ for 3 h at 37°C according to the manufacturer’s instructions (NEB, United States). The deglycosylated and untreated samples were analyzed by SDS-PAGE. For substrate specificity, chitosan with different DA (85, 90, and 95%), colloidal chitin, xylan, and microcrystalline cellulose were used as substrate. The kinetic parameters were detected using different concentrations of chitosan with 90% DA (1, 1.5, 2, 2.5, 3, 4, 5, 6, and 8 mg/ml) as substrate. The values of *V*_*max*_ and *K*_*m*_ were calculated by program Graft.

### Characterization of CsnBm

The optimal pH of CsnBm was assayed in different 50 mM buffer from pH 3.5 to 7 (acetic acid-sodium acetate for pH 3.5 to 6 and Na_2_HPO_4_-NaH_2_PO_4_ for pH 6.5 to 7.0). The relative activity at different pH was calculated by setting pH 5.5 as 100%. For pH stability, CsnBm was incubated at 25°C in 50 mM buffer with different pH from 4.0 to 11.0 for 6 h (acetic acid-sodium acetate for pH 3.0 to 6.0, Na_2_HPO_4_-NaH_2_PO_4_ for pH 6.0 to 8.0, Tris-HCl for pH 7.0 to 9.0, and Gly-NaOH for pH 9.0 to 10.0), and then the residual activity was determined. The enzyme activity of the sample treated with distilled water was considered as 100%, and other values were recorded as a percentage of the highest value. All measurements were carried out in triplicate.

The optimal temperature of CsnBm was measured at different temperatures from 30 to 70°C. The relative activities at different temperature were calculated by setting 55°C as 100%. The thermal stability of CsnBm was studied by incubating at temperatures from 40 to 70°C for 20, 40, and 60 min, and the residual activity was determined at pH 5.5 and 55°C. The residual activity was calculated by taking the activity of non-heated CsnBm as 100%. All measurements were carried out in triplicate.

The effect of different metal ions on the stability of CsnBm was analyzed by incubating enzyme samples for 4 h at room temperature in 50 mM Tris-HCl buffer (pH 7.0), containing 1 and 5 mM of Ca^2+^, Mg^2+^, Na^+^, K^+^, Li^+^, Zn^2+^, Mn^2+^, Co^2+^, Hg^2+^, Ag^+^, and Fe^2+^. The residual activity was determined as described previously.

### Hydrolytic Pattern of CsnBm

The hydrolytic properties of CsnBm were determined and analyzed according to a previous study (Guo et al., 2019). The 0.5% (w/v) colloidal chitosan with 90% DA, (GlcN)_2_, (GlcN)_3_, (GlcN)_4_, (GlcN)_5_, and (GlcN)_6_ were used as substrate to investigate the hydrolytic properties of CsnBm. Purified CsnBm was added to 0.5% (w/v) colloidal chitosan (sodium acetate buffer pH 5.5, 0.2 mM) and 0.3% (w/v) COSs (dissolved in distill water), and then incubated at 55°C for 2 h. Samples withdrawn at different times were immediately incubated at 90°C for 10 min and centrifuged at 10,000 × *g* for 5 min. The samples withdrawn at different times were analyzed by thin-layer chromatography (TLC) method. Samples were spotted on a TLC plate, developed in isopropanol:water:ammonium hydroxide (15:1:7.5, v/v) as solvent, and sprayed with 0.2% ninhydrin (dissolved in ethanol). The hydrolysis products were visualized by heating the plate at 100°C for 10 min.

### Production of Chitosan Oligosaccharides by CsnBm

During the process of production of COSs by CsnBm, the colloidal chitosan with 90% DA was used as substrate. The supernatant of crude CsnBm from 50-L high-density fermentation was used for the preparation of COSs. Reactions were carried out in a 50-ml flask containing 10 ml of different concentrations of colloidal chitosan (1, 2, 3, and 4%, w/v) with different amounts of the crude CsnBm (3, 6, 9, 12, and 15 U/ml). The flasks were incubated at 55°C and 100 rpm for 1 h and the reaction was stopped by incubating at 90°C for 10 min, and then 1 M NaOH was added to adjust the pH to 8.5 and finally centrifuged at 10,000 × *g* for 5 min. The hydrolytic products were analyzed by TLC and high-performance liquid chromatography (HPLC) method. The HPLC system (Thermo Fisher Scientific, United States) was equipped with a refractive index detector and a Zorbax carbohydrate analysis column (4.6 × 250 mm, 5 μm) (Agilent, United States). The mobile phase was composed of acetonitrile and water (70:30, v/v) and the flow rate was 0.8 ml/min. The concentrations of different COSs were quantified by integrating peak areas according to the respective standard curve (Qin et al., 2018b). The method for calculation of total COSs yield is provided in the Supplementary Material. Furthermore, the concentrations of different COSs and total COSs yield at different reaction time were studied. The reaction was carried out in a 500-ml flask containing 200 ml of 4% colloidal chitosan (w/v) with 9 U/ml of CsnBm. The flasks were incubated at 55°C and 100 rpm, the reaction samples (1 ml) were withdrawn at 5, 10, 15, 20, 25, 30, 40, 50, and 60 min. The methods for sample treatment and analysis were the same as abovementioned. The effects of different CsnBm additions on the production of the same COS were analyzed. Experiments were conducted in triplicate, and measurements were presented with their means and SD. Data were subjected to one-way ANOVA analysis by SPSS (version 24.0) and Duncan’s multiple range tests (*p* < 0.05) to compare the mean value of different treatments.

## Results and Discussion

### Gene Cloning and Bioinformatics Analysis

Sequence analysis showed that the open reading frame of CsnBm was 837 bp (Accession No, OK172330), which encoded 278 amino acid residues. The molecular weight and the theoretical pI of the deduced amino acid sequence (named CsnBm) were 31.3 kDa and 7.68, respectively, predicted by ProtParam. The first 36 amino acid residues of CsnBm were signal peptide. The results of NCBI blastp revealed that CsnBm shared 98.1% identity to chitosanase from *B. mojavensis* UCMB5075 (Accession No. WP_168747460.1), followed by chitosanase *Bacillus halotolerans* (96.3% identity, Accession No. WP_059336147.1). Multiple alignment of CsnBm with previous reported chitosanases showed that Glu19 and Asp35 were catalytic active sites. In addition, a conservative motif (DGRGYT) was found among these different chitosanases (Supplementary Figure 1).

The tertiary structure of CsnBm was obtained by homology modeling using chitosanase from *B. subtilis* MY002 as template (PDB deposition: 7c6c.1). The identity of tertiary structure between CsnBm and chitosanase from *B. subtilis* MY002 was 91.74%, and the overall structure of CsnBm is also close to previously reported GH46 members (Supplementary Figure 2A). The molecular structure of CsnBm can be divided into upper and lower domains, which contains nine α-helices and two β-strands (Supplementary Figure 2B). The predicted substrate binding region of CsnBm was a closed tunnel that is different from other GH46 chitosanases with open clefts (Marcotte et al., 1996; Saito et al., 1999; Lyu et al., 2015; Wang et al., 2020). The substrate binding region of CsnBm was highly negatively charged that is suitable for binding the cationic chitosan (Supplementary Figure 2C). The hydrogen bonds were the main force to stabilize the network between (GlcN)_6_ and substrate binding region of CsnBm. As shown in Supplementary Figure 2D, several residues play an important role in binding substrate, which is similar to previous studies (Takauka et al., 2014; Li et al., 2021).

### Heterologous Expression of CsnBm in *Pichia pastoris* X33

The 726-bp PCR product (named *csnbm*) without the native signal sequence was integrated in frame with the *Saccharomyces cerevisiae* α-factor secretion signal sequence under the control of the AOX1 promoter in plasmid pPICZαA to obtain the expression vector pPICZαA-*csnbm*. The expression vector pPICZαA-*csnbm* was linearized and transformed into *P. pastoris* X33. After transformation, many colonies were formed on the YPDZ plates. For initial screening, a total of 120 recombinant colonies were screened. Among these 120 recombinant colonies, six recombinant colonies (named recombinant strain X33-C1 to X33-C6) with higher activity (from 13 to 21 U/ml) were selected and cultivated in shaking flask. The results of shaking flask fermentation are shown in Supplementary Figure 3. The recombinant strain X33-C3 exhibited the highest activity (121 U/ml) among these six recombinants. Therefore, the recombinant strain X33-C3 was chosen for high cell density fermentation.

**FIGURE 1:**
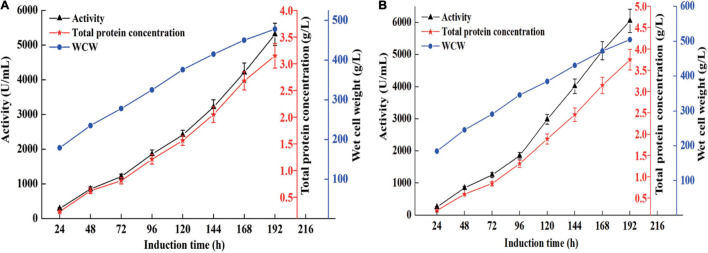
The chitosanase activity, total protein concentration, and cell growth of the recombinant strain X33-C3 during fed-batch fermentation in 7-L **(A)** and 50-L **(B)** bioreactors.

High cell density fermentation was carried out in 7- and 50-l bioreactors. As shown in Figure 1A, the maximum activity and total protein concentration produced by recombinant strain X33-C3 in the 7-l bioreactor reach 5,310 U/ml and 3.15 g/L, respectively. Meanwhile, the maximum activity and total protein concentration produced by recombinant strain X33-C3 in the 50-l bioreactor were 6,052 U/ml and 3.75 g/l, respectively (Figure 1B). In this study, the maximum activity of recombinant strain X33-C3 in the 50-l bioreactor was about 50-fold to that in shake flask cultivation (121 U/ml). Based on the results of this and previous studies, we can make a conclusion that high cell density fermentation is an effective method to improve the production of chitosanase in *P. pastoris*. The production of recombinant chitosanase from *Bacillus amyloliquefaciens* (*B. amyloliquefaciens*) is almost improved by 35.91-fold from the shake flask to the 5-l bioreactor (Luo et al., 2020). The study of Zhou R. et al. (2020) also reported that the enhancement of recombinant chitosanase from *Aspergillus oryzae* NKY2017 is almost 70-fold by high cell density fermentation.

**FIGURE 2:**
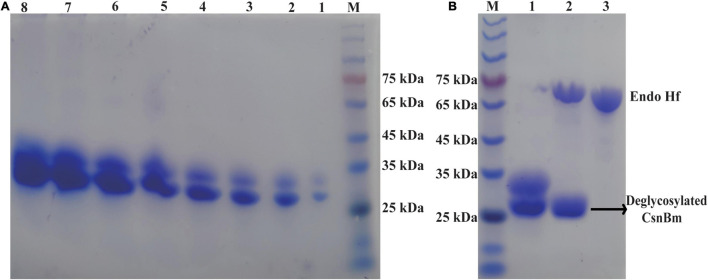
SDS-PAGE analysis of CsnBm. **(A)** Supernatant from different induction time in 50-L bioreactors. M, marker, lanes 1–8 represent supernatant from 24 to 192 h. **(B)** Purified and EndoH_*f*_ treatment CsnBm. M marker, lane 1: purified CsnBm, lane 2: Deglycosylated CsnBm and Endo H_*f*_, lane 3: Endo H_*f*_.

According to the previous studies, we found that *E. coli* is the most favorite host for heterologous expression of chitosanase, followed by *P. pastoris*. Compared with *E. coli*, *P. pastoris* has some advantages such as extracellular expression and very low secretion levels of endogenous proteins, which are suitable for large-scale preparation of recombinant chitosanase. In this study, the production of CsnBm was 3.75 g/l and the recombinant CsnBm is the main protein of supernatant (Figure 2A). The result of this study is similar with previous works; some recombinant chitosanases were overexpressed at high level during methanol induction in *P. pastoris*. The expression level of chitosanases from *Mitsuaria* sp. 141, *B. amyloliquefaciens*, *Aspergillus oryzae* NKY2017, and *Streptomyces* sp. N174 is almost 1.6, 4.5, 3.1, and 8.5 g/l, respectively (Peng et al., 2013; Ding et al., 2019; Luo et al., 2020; Zhou R. et al., 2020). Furthermore, all of these recombinant chitosanases are approximately free from contaminating proteins, which facilitates downstream processing.

### Purification, Kinetic Parameters, and Substrate Specificity

The recombinant CsnBm from the culture supernatant was purified by Ni^2+^-NTA resin chromatography (Supplementary Table 1). After ultrafiltration and affinity chromatography, the specific activity of CsnBm was 2,663 U/mg. The purified CsnBm showed two bands on SDS-PAGE, which were approximately 33 and 27 kDa, respectively (Figure 2A). The band with 33 kDa was larger than the calculated molecular mass of CsnBm that may probably due to glycosylation. CsnBm was found to have one N-glycosylation site (N20GTT) by N-glycosylation site analysis (NetNGlyc 1.0 Server).^1^ After deglycosylation using EndoH_*f*_, there was only a single band about 27 kDa, suggesting that the 33-kDa band was a glycoprotein (Figure 2B).

Kinetic parameters of CsnBm were determined, and the values of *K*_*m*_ and *V*_*max*_ of CsnBm were 0.71 mg/ml and 2,802 μM/min/mg, respectively (Supplementary Table 2). The CsnBm with very low *K*_*m*_ and high *V*_*max*_ revealed that it has high substrate affinity and catalytic efficiency. CsnBm exhibited the highest activity toward colloidal chitosan with 90% DA, followed by colloidal chitosan with 95 and 85% DA, respectively (Table 1). Similar to other chitosanases from the GH46 family, CsnBm exhibited no activity toward microcrystalline cellulose, xylan, and colloidal chitin.

**TABLE 1:** The substrate specificity of CsnBm.

**Substrate**	**Relative activity (%)**
Colloidal chitosan with 85% DA	91
Colloidal chitosan with 90% DA	100
Colloidal chitosan with 95% DA	93
Microcrystalline cellulose	ND
Colloidal chitin	ND
Xylan	ND

*ND represents the enzyme activity was not detected.*

### Characterization of CsnBm

**FIGURE 3:**
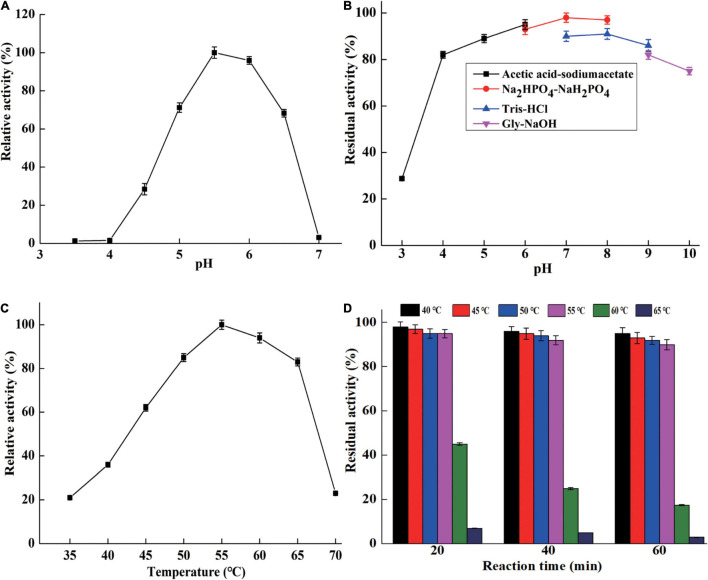
The characterization of purified CsnBm. Optimum pH **(A)**, pH stability **(B)**, optimum temperature **(C)**, and thermostability **(D)**.

CsnBm exhibited maximum activity at pH 5.5 and remained active at a pH range from 5 to 6.5 (Figure 3A). The optimal pH of CsnBm is similar to chitosanase from *Gynuella sunshinyii* (*G. sunshinyii*) and *Aspergillus fumigatus* (*A. fumigatus*) CJ22-326 (Qin et al., 2018a; Zhou J. L. et al., 2020). CsnBm was stable from pH 4.0 to 9.0, and the residual activity was above 80% after 4 h of incubating at 25°C (Figure 3B). The solubility of chitosan is a key factor on hydrolysis efficiency of chitosan. CsnBm was active and stable from pH 4.5 to 6.0, which is suitable for production of COSs, since chitosan has better solubility below pH 6.0. The optimal temperature of CsnBm was 55°C and the relative activities from 50 to 65°C were above 80% (Figure 3C). CsnBm was stable from 45 to 55°C, the residual activities of CsnBm were above 90% after heat treatment for 20 to 60 min (Figure 3D). CsnBm decreased dramatically when the temperature was higher than 55°C, and the residual activities of CsnBm were only 45, 25, and 15%, respectively, after 20 to 60 min incubation at 60°C. For 65°C, CsnBm exhibited only 7% residual activity after 20 min incubation. The thermal stability of CsnBm is better than chitosanase Csn-SH from *Bacillus atrophaeus* (*B. atrophaeus*), BaCsn46A and BaCsn46B from *B. amyloliquefaciens*, and CsnQ from *Bacillus* sp. Q1098 (Qin et al., 2018b; Luo et al., 2020; Ma et al., 2020; Cui et al., 2021). The possible reason for CsnBm with better thermal stability was N-glycosylation, which is a common post-translational modification in *P. pastoris* (Bretthauer and Castellino, 1999). Some previous works revealed that glycosylation is helpful for the thermal stability and catalytic activity of recombinant proteins (Fonseca-Maldonado et al., 2013; Cheng et al., 2015). The temperature properties of CsnBm revealed that it is suitable for preparation of COSs at 55°C. Reaction temperature at high temperature is helpful for improving hydrolysis efficiency and reducing risk of microbial contamination (Botha et al., 2018).

CsnBm was activated by Ca^2+^, Zn^2+^, Mg^2+^, and Mn^2+^, which were 1.09- to 1.8-fold than that of the control, when the concentration of these metal ions was 1 or 5 mM (Table 2). CsnBm was inhibited by Fe^2+^ and Cu^2+^, especially when Fe^2+^ was 5 mM, and the residual activity was only 6% (Table 2). K^+^ and Co^2+^ had little effect on the activity of CsnBm. Based on the results of this study and some previous researches, we found that metal ions are activators or inhibitors mainly depending on the specific target chitosanases. In some previous studies, Mn^2+^ has a positive effect on improving the activity of chitosanase. For example, the activities of chitosanase from *A. fumigatus* CJ22-326, *Bacillus* sp. MD-5, and *Chromobacterium violaceum* are improved by 3. 0-, 1. 2-, and 2.8-fold, respectively, in the presence of Mn^2+^ (Azevedo et al., 2020; Yang et al., 2020; Zhou J. L. et al., 2020). However, the chitosanase from *Streptomyces roseolus*, *Acinetobacter calcoaceticus* TKU024, and *Serratia marcescens* TKU011 are inhibited by Mn^2+^ (Wang et al., 2008, 2011; Jiang et al., 2012). In this study, the relative activity of CsnBm was increased by 10% in the presence of Zn^2+^, similar to the chitosanases from *Staphylococcus capitis* and *Streptomyces niveus* (Sun et al., 2018; Chen et al., 2021). On the contrary, the chitosanase from *Aspergillus* sp. W-2 and *Aquabacterium* sp. A7-Y is inhibited by Zn^2+^ (Zhang et al., 2015; Wang et al., 2021).

**TABLE 2:** Effects of different metal cations on CsnBm activity.

**Metal cations**	**Residual activity (%)**	
	**1 mM**	**5 mM**
Control	100	100
Co^2+^	92	86
Cu^2+^	62	30
Fe^2+^	9	6
Na^+^	96	95
Ca^2+^	112	109
K^+^	96	93
Mn^2+^	180	153
Mg^2+^	115	112
Zn^2+^	121	111

### Hydrolytic Pattern of CsnBm

**FIGURE 4:**
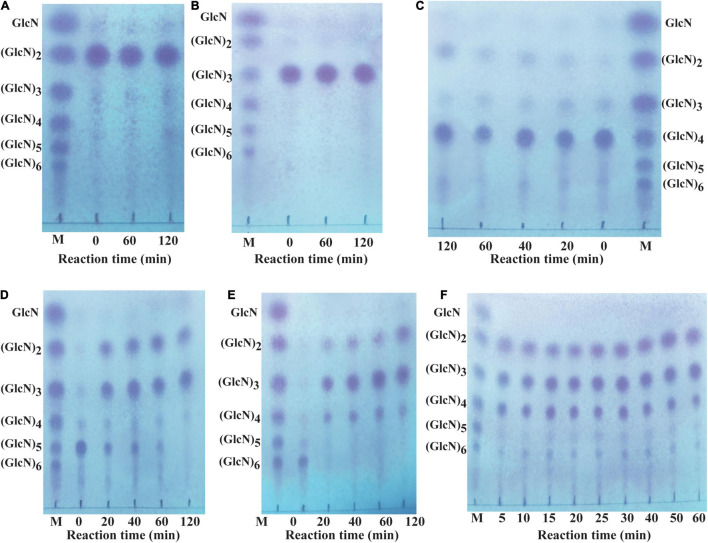
Analysis of the hydrolytic process of CsnBm toward COSs and 0.5% colloidal chitosan. Chitobiose **(A)**, Chitotriose **(B)**, Chitotetraose **(C)**, Chitopentaose **(D)**, Chitohexaose **(E)**, and 0.5% colloidal chitosan **(F)**. M, marker sugars containing GlcN, (GlcN)_2_, (GlcN)_3_, (GlcN)_4_, (GlcN)_5_, and (GlcN)_6_.

Different COSs and 0.5% colloidal chitosan with 90% DA were selected as substrate to analyze the hydrolytic pattern of CsnBm. CsnBm exhibited no activity toward (GlcN)_2_ and (GlcN)_3_; even the reaction time prolonged to 2 h (Figures 4A,B). As shown in Figure 4C, a little (GlcN)_4_ is cleaved and converted to (GlcN)_2_ after 2 h incubation, indicating that CsnBm hardly hydrolyzed the β-1,4 linkage in (GlcN)_4_. CsnBm showed high efficiency for (GlcN)_5_ and (GlcN)_6_, and most of (GlcN)_5_ was cleaved into (GlcN)_2_ and (GlcN)_3_ after 20 min incubation (Figure 4D). CsnBm completely hydrolyzed (GlcN)_6_ to yield (GlcN)_2_, (GlcN)_3_, and (GlcN)_4_ only after 20 min incubation (Figure 4E). The products of 0.5% colloidal chitosan hydrolyzed by CsnBm are shown in Figure 4F. The main products were (GlcN)_2_, (GlcN)_3_, and (GlcN)_4_, incubated for 5 to 60 min.

No GlcN was detected during the hydrolytic process of COSs and 0.5% colloidal chitosan in this study. These results showed that CsnBm is an endo-type chitosanase. Furthermore, based on the hydrolytic process of (GlcN)_6_, we deduced that CsnBm may have two binding and cutting modes. One cutting mode of CsnBm was the “4 + 2” splitting mode that CsnBm hydrolyzed (GlcN)_6_ to produce (GlcN)_2_ and (GlcN)_4_. The other was the “3 + 3” splitting mode that hydrolyzed (GlcN)_6_ to produce (GlcN)_3_. A previous study found that chitosanase interacts with (GlcN)_6_ in three ways, which are related to subsites −2 to + 4, −3 to + 3, and −4 to + 2, respectively (Pechsrichuang et al., 2018). The hydrolytic pattern of CsnBm is similar to previous reported chitosanases. The chitosanases from *B. amyloliquefaciens*, *G. sunshinyii*, *Bacillus* sp. MD-5, and marine *Bacillus* sp. exhibit no activity toward (GlcN)_2_ and (GlcN)_3_ and the final hydrolysis products are mainly composed of (GlcN)_2_, (GlcN)_3_, and (GlcN)_4_ (Qin et al., 2018a,b; Ma et al., 2020; Yang et al., 2020).

### Production of Chitosan OligosaccharidesCOSs by CsnBm

**FIGURE 5:**
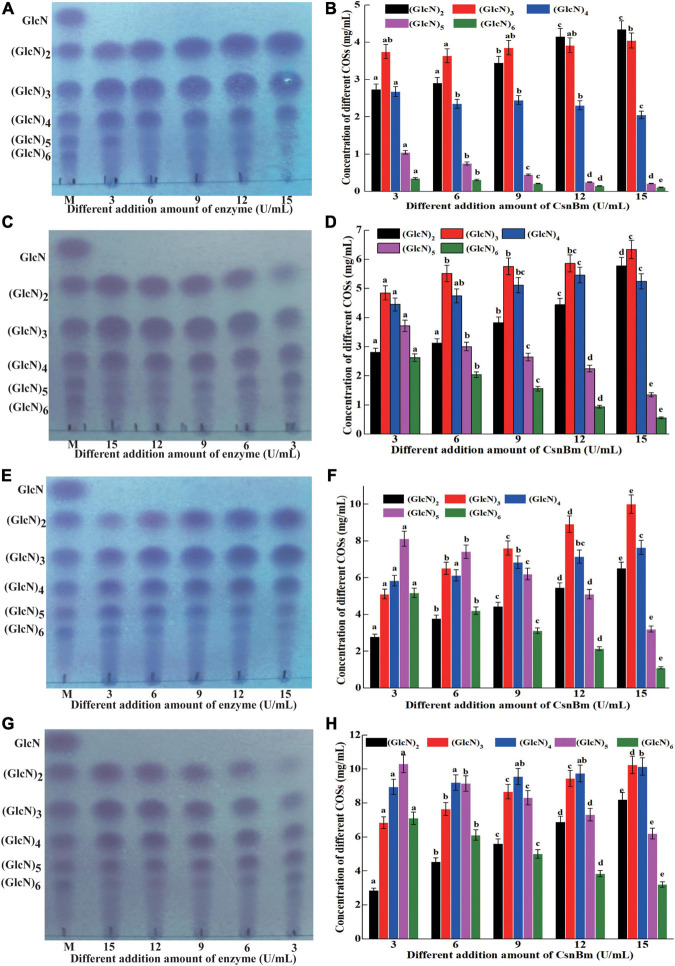
Analysis of hydrolysates from different concentration of colloidal chitosan addition with different amounts of CsnBm. TLC analysis of hydrolysates from 1% **(A)**, 2% **(C)**, 3% **(E)**, and 4% colloidal chitosan **(G)**. M, marker sugars containing GlcN, (GlcN)_2_, (GlcN)_3_, (GlcN)_4_, (GlcN)_5_, and (GlcN)_6_. HPLC analysis of hydrolysates from 1% **(B)**, 2% **(D)**, 3% **(F)**, and 4% colloidal chitosan **(H)**. The effects of different CsnBm additions on the production of the same COS were analyzed. Different lowercase superscripts in the columns with same color indicated statistical difference (*p* < 0.05). Experiments were conducted in triplicate, and measurements were presented with their means and SD. Data were subjected to one-way ANOVA analysis by SPSS (version 24.0) and Duncan’s multiple range tests (*p* < 0.05) to compare the mean value of different treatments.

Previous reports have shown that COSs with different degrees of polymerization (DP) exhibit different biological activities (Li et al., 2016; Yuan et al., 2019). Li et al. (2012) found that COS with DP 2 to 6 showed obvious scavenging activity against hydroxyl radical and COS with low DP exhibits better reducing power. In addition, Li et al. (2014) also investigated the antimicrobial activity against *Staphylococcus aureus* of COSs with DP 2 to 12 and found that the COS exhibits antibacterial activity with a DP of at least 5. Previous studies found that COSs with DP 2 to 7 could improve the chilling tolerance of wheat seedlings and COS with DP7 shows the best effect (Zou et al., 2017). Therefore, it is necessary to investigate the process of preparation of COSs by CsnBm. The hydrolysis reactions were carried out with different concentrations of colloidal chitosan (1 to 4%, w/v) and different amounts of CsnBm (3, 6, 9, 12, and 15 U/ml). The results of different hydrolysis reactions are shown in Figure 5. The hydrolysis of 1% colloidal chitosan mainly produced (GlcN)_2_, (GlcN)_3_, and (GlcN)_4_ as final products (Figures 5A,B). As shown in Figures 5C,D, the hydrolysates of 2% colloidal chitosan mainly include (GlcN)_2_, (GlcN)_3_, (GlcN)_4_, and (GlcN)_5_, when the amounts of CsnBm were 3, 6, and 9 U/ml, respectively. As the amount of CsnBm increased to 12 and 15 U/ml, the hydrolysates were mainly composed of (GlcN)_2_, (GlcN)_3_, and (GlcN)_4_. For 3% colloidal chitosan addition with 3, 6, and 9 U/ml of CsnBm, the end products mainly contained (GlcN)_2_, (GlcN)_3_, (GlcN)_4_, (GlcN)_5_, and (GlcN)_6_ (Figures 5E,F). With the increased addition of CsnBm to 12 and 15 U/ml, the end products were mainly composed of (GlcN)_2_, (GlcN)_3_, (GlcN)_4_, and (GlcN)_5_. The hydrolysates 4% colloidal chitosan added with 3 and 6 U/ml were predominantly composed of (GlcN)_3_, (GlcN)_4_, (GlcN)_5_, and (GlcN)_6_ (Figures 5G,H). As the amount of CsnBm increased to 9, 12, and 15 U/ml, the hydrolysis of 4% colloidal chitosan produced (GlcN)_2_, (GlcN)_3_, (GlcN)_4_, and (GlcN)_5_ as the main end products (Figures 5G,H). The HPLC charts of all reactions are shown in Supplementary Figure 4. The total COSs yields of all reactions were above 91.2% (Supplementary Figure 5).

**FIGURE 6:**
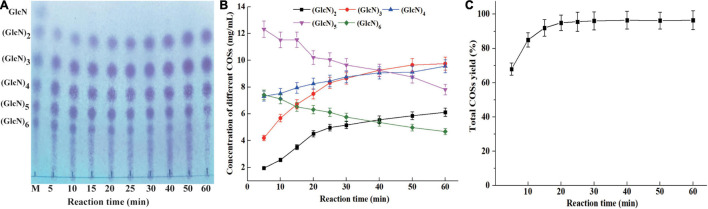
Analysis of hydrolysates of 4% colloidal chitosan with 9 U/ml crude CsnBm at different reaction times. **(A)** TLC analysis of hydrolysates from different reaction time. M, marker sugars containing GlcN, (GlcN)_2_, (GlcN)_3_, (GlcN)_4_, (GlcN)_5_, and (GlcN)_6_. **(B)** The concentrations of COSs at different reaction time. **(C)** Total COSs yield of 4% colloidal chitosan at different reaction time.

The concentration of different COSs and total COSs yields under different reaction times with 4% colloidal chitosan addition with 9 U/ml CsnBm were further studied. As shown in Figures 6A,B, the COSs mixture mainly include (GlcN)_2_, (GlcN)_3_, (GlcN)_4_, (GlcN)_5_, and (GlcN)_6_ after 5 min reaction and the total COSs yield was 68.2% (Figure 6C). After 10 min of reaction, the total COS yield increased from 68.2 to 85.3%, and the concentrations of (GlcN)_2_, (GlcN)_3_, (GlcN)_4_, (GlcN)_5_, and (GlcN)_6_ increased gradually. The total COSs yield reached 92.3% when the reaction time was 15 min and the concentrations of (GlcN)_2_, (GlcN)_3_, (GlcN)_4_, (GlcN)_5_, and (GlcN)_6_ were 3.52, 6.72, 7.95, 11.53, and 6.52 g/L (Figure 6B). During the hydrolysis time from 20 to 60 min, the COSs with higher DP (6 and 5) were gradually cleaved into (GlcN)_2_, (GlcN)_3_, and (GlcN)_4_ (Figures 6A,B). The total COSs yields of reactions from 20 to 60 min were in the range from 95.1 to 96.5% (Figure 6C). The HPLC charts of all reactions are shown in Supplementary Figure 6. CsnBm exhibited high hydrolytic activity on chitosan and the above results demonstrated that CsnBm is suitable for the controllable production of COSs.

Up to now, many chitosanases have been used to prepare COSs (Table 3). Zhou J. L. et al. (2020) reported that the total COSs yield reaches 97.29% after 8 h reaction with 2% chitosan addition with 30 U/ml of *A. fumigatus* CJ22-326 chitosanase. The bifunctional chitosanase PbCsn8 from *Paenibacillus barengoltzii* could cleave 5% chitosan into (GlcN)_2_, (GlcN)_3_, and (GlcN)_4_ in the presence of 5 U/ml PbCsn8 after 4 h reaction and the total COSs yield is 79.3% (Jiang et al., 2021). The work of Qin et al. (2018b) found that the total COSs yield of 3% chitosan is 86.85% after the addition of 100 U/g chitosanases from *B. amyloliquefaciens* for 3 h hydrolysis reaction. In this study, after 15 min reaction in the presence of 9 U/ml CsnBm, the total COSs yield of 4% chitosan was 92.3%, which is more efficient than previously reported chitosanases.

**TABLE 3:** Enzymatic conversion of chitosan to COSs by chitosanases.

**Microorganism source**	**Amount of enzyme (U/ml)**	**Concentration of chitosan (%)**	**Reaction time (Min)**	**Total COSs yield (%)**	**Major products**	**References**
*A. fumigatus* CJ22-326	30	2	480	97.3	(GlcN)_2_ to (GlcN)_6_	Zhou J. L. et al. (2020)
*P. barengoltzii*	5	5	240	79.3	(GlcN)_2_ to (GlcN)_4_	Jiang et al. (2021)
*B. amyloliquefaciens*	3	3	180	86.9	(GlcN)_2_ to (GlcN)_6_	Qin et al. (2018b)
*B. atrophaeus* BSS	20	4	40	80.6	(GlcN)_2_ to (GlcN)_6_	Cui et al. (2021)
*B. mojavensis* SY1	9	4	15	92.3	(GlcN)2 to (GlcN)6	This study

## Conclusion

In this study, the high-efficiency chitosanase CsnBm from *B. mojavensis* SY1 was high-level heterologously expressed and biochemically characterized. The maximum activity and total protein concentration of CsnBm were 6,052 U/ml and 3.75 g/l, respectively. The purified CsnBm was most active at 55°C and pH 5.5. The *K*_*m*_ and *V*_*max*_ of CsnBm were 0.71 mg/ml and 2,802 μM/min/mg, respectively. CsnBm exhibited high efficiency hydrolysis of chitosan to produce COSs. The excellent properties and high-level production of CsnBm will provide a basis for its application in industrial-scale preparation of COSs.

## Data Availability Statement

The data presented in the study are deposited in the NCBI, accession number OK172330.

## Author Contributions

JW contributed to gene clone, construct recombinant strain, and bioinformatics analysis of CsnBm. XL contributed to analysis of COSs by TLC and HPLC. HC contributed to the analysis of the hydrolytic properties of CsnBm. BL contributed to high-density fermentation and purification of CsnBm. LZ contributed to experiment planning. All authors contributed to the article and approved the submitted version.

## Conflict of Interest

The authors declare that the research was conducted in the absence of any commercial or financial relationships that could be construed as a potential conflict of interest.

## Publisher’s Note

All claims expressed in this article are solely those of the authors and do not necessarily represent those of their affiliated organizations, or those of the publisher, the editors and the reviewers. Any product that may be evaluated in this article, or claim that may be made by its manufacturer, is not guaranteed or endorsed by the publisher.

## Footnotes

1
http://www.cbs.dtu.dk/services/NetNGlyc/
